# Photonic Hooks Generated by a Concave Micro-Cylinder Based on Structure-Constrained Functions

**DOI:** 10.3390/mi13091434

**Published:** 2022-08-30

**Authors:** Jialing Zhang, Guoxia Han, Ze Yang, Shuyue Xie, Kaiyun Zhan

**Affiliations:** College of Science, China University of Petroleum (East China), Qingdao 266580, China

**Keywords:** photonic hook, structure-constrained function, effective length, bending angle

## Abstract

Owing to its crooked trajectory and small full width at half-maximum, photonic hook (PH) has attracted wide attention since its inception and experimental confirmation. However, the present generation and regulation of PH are mostly dependent on the breaking of the symmetry of the system composed of the incident light and the regular structure particles, which inevitably limits the research of PH. In this work, the PH of the irregular particles is demonstrated with the help of a structure-constrained function (SCF). By varying the coefficients of the function, characteristic parameters of the PH, such as the bending angle, the effective length and the bending direction, can be effectively modulated. Meanwhile, high-quality PHs with a bending angle of up to 46° and an effective length of up to 11.90*λ*, as well as PHs with three bends, can be obtained using this method. The formation mechanism of the PH is revealed by simulating the distribution of the field intensity with the finite element method and analyzing with ray optics. This is the first time that we introduce a function into the investigation of PH, paving a new way for a more interesting exploration of PH.

## 1. Introduction

Photonic nanojet (PNJ) is a jet structure that is generated at the shadow side of an illuminated dielectric micro-particle. It was first theoretically predicted by Adler and Lock et al. from the perspectives of ray optics and electromagnetic waves, respectively, in 1997 and 2000 [1,2], and verified by Chen et al. with the high-resolution finite-difference-time-domain-method (FDTD) numerical solutions of Maxwell’s equations in 2004 [3]. Owing to its high local area and narrow beam waist, PNJ has attracted much attention [4,5,6,7,8,9,10], which has wide applications such as optical storage [11,12], micro-nano manipulation [13,14], super-resolution imaging [15,16,17,18], and optical detection [4,10,19,20]. It has been known that the PNJ propagates in a straight line, and most research focuses on how to extend its length [21,22] and reduce its waist [23,24]. In 2018, I. V. Minin and O. V. Minin first introduced the photonic hooks (PHs) [25] which is a kind of subwavelength locally curved beam, indicating that PNJ can also propagate along a curvilinear trajectory. This finding greatly widens the research field of PNJ, stimulating the emergence of acoustic hook [26] and plasma hook [27,28,29].

To achieve a tunable length and bending angle of PH, several generation approaches [30,31,32] have been proposed. One of the approaches is asymmetry illumination. In 2016, Wang’s group reported the curved PNJ by the asymmetry illumination of the beam on micro-particle [33], which was essentially later proposed PH. Another approach is utilizing asymmetry materials. In 2018, Gu et al. studied the formation of PH with a Janus micro-cylinder composed of two half-cylinder with different materials [34]. The bending angle of the PH can be modulated flexibly by rotating the Janus micro-cylinder relative to the central axis. In addition to above-mentioned approaches, PH can be also obtained by asymmetry structure. In 2020, based on the specular reflection of a tilted flat mirror, Geints et al. regulated the bending angle of the PH by adjusting the tilt degree of the mirror [35]. Meanwhile, Liu and co-workers proposed that adding a metal baffle in front of the light incident surface of the ellipsoid structure can be used to regulate the bending direction of the PH [36]. This method was supported by Ang et al., who pointed out that varying the refractive index and thickness of the metal baffle in front of the micro-trapezoidal structure can influence the features of PH [37]. To quantitatively characterize the bending angle of PH, Gu’s group specifically defined the position of “inflection point” for the first time in 2021 [38], which was the boundary point between the region of rapid change (RRC) and region of slow change (RSC) of the PH. So far, the present generation and regulation of PH are mostly dependent on the breaking of the symmetry of the system composed of the incident light and the regular structure particles, such as digging columns, holes, triangles, and rectangles [39,40,41,42], which inevitably limits the research of PH.

Considering the above-mentioned issues, PH generated by the irregular structure is not yet well understood. For the first time, we introduced a structure-constrained function (SCF) into constraining an irregular concave micro-cylinder structure to achieve tunable PHs. SCF is used to describe the boundary of the irregular structure of the particle, thus influencing the length, the bending angle, and the bending direction of the PH. An adjustable PH can be facilely obtained by changing coefficients of SCF. This paper is organized as follows: the model, the function, and the PH parameters are given in Section 2; in Section 3, we investigated the influence of different irregular structures on the generation of PH—the underlying principle of adjusting the depression structure of the micro-cylinder to generate PHs is revealed and the effect of the function’s coefficients is also analyzed in detail; finally, conclusions are drawn in Section 4.

## 2. Models and Method

**Figure 1:**
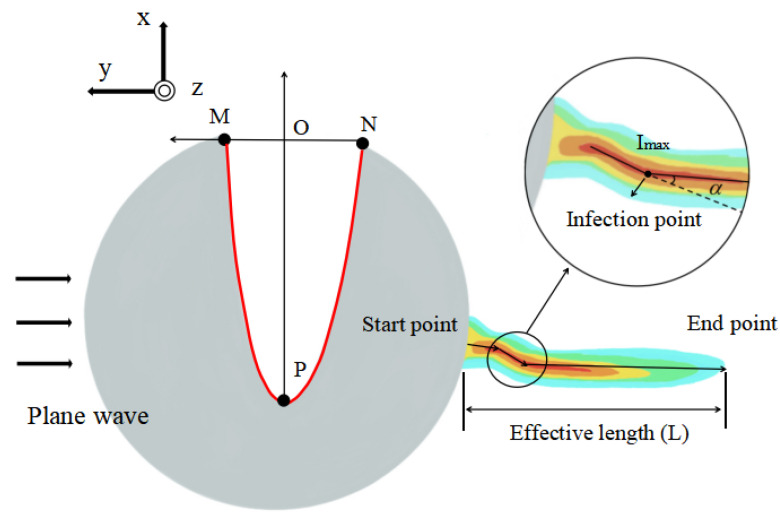
A 2D cross-section. The red curve represents the SCF. The coordinates of *M*, *N*, and *P* are (0, *a*), (0, −*b*), and (−*c*, 0), respectively. The maximum electric field intensity of the optical hook is *I_max_*, and the boundary intensity of the optical hook is *I_max_/*$\sqrt{e}$. The effective length *L* is the distance from the start point to the end point, and the bending angle is *α*.

As shown in Figure 1, a monochromatic plane wave, polarized along the *x* axis and propagating negatively along the *y* axis, is incident perpendicularly on a micro-cylinder with a concave in a vacuum. The PH is generated at the shadow side of the cylinder and three characteristic parameters—*I_max_*, *L*, and *α*—are defined. The maximum electric field intensity of the optical hook is defined as *I_max_*, and the boundary intensity is *I_max_/*$\sqrt{e}$. Along the main lobe of the optical hook, the starting point and the end point of the electric field intensity reaching the boundary intensity are specified as the “start point” and the “end point”, respectively. The effective length *L* is the projection distance of the optical hook along the *y* axis from the start point to the end point. The bending angle of the optical hook is defined as *α*. It is specified that the bending angle is positive when the PH bends to the right. Otherwise, the bending angle is negative. We introduced an asymmetric parabola-like function with a single intersection with the coordinate axis to describe the boundary of the concave, i.e., SCF, as shown by the red curve in Figure 1.

The specific expression of the function applied here is expressed in the following equation,

*x* = *Ay*^2^ + *By* + *C* + *Dxy* (1)

where *A*, *B*, *C*, and *D* are coefficients of the SCF. The three points *M*, *N*, and *P* represent the three vertices of the depression, which are the upper intersection, the lower intersection, and the vertex, with their coordinates of (0, *a*), (0, −*b*), and (−*c*, 0), respectively. The relationships among coordinates of *a*, *b*, and *c*, and coefficients of *A*, *B*, *C*, and *D* are expressed as the following formulae with the specific derivation given in Appendix A.

(2) $$A=\frac{c}{ab}$$

(3) $$B=-\frac{(a-b)c}{ab}$$

(4) C=−c

(5) $$D=-\frac{\left(a-b\right)}{ab}$$

**Figure 2:**
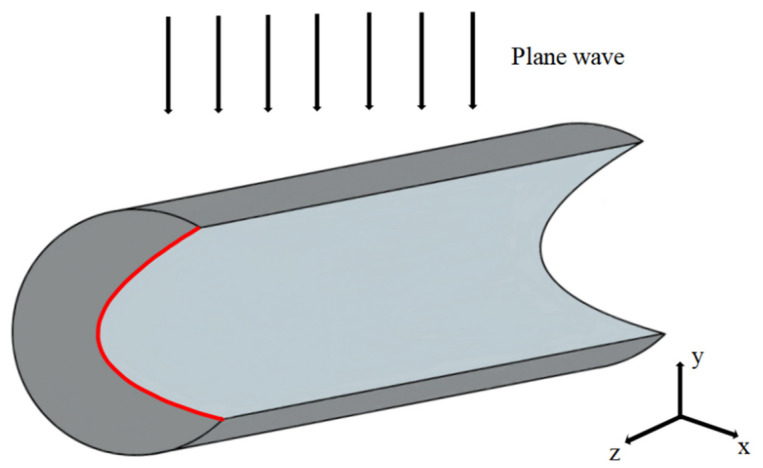
3D schematic view. The red curve represents the SCF.

According to these relations, it can be seen that the positions of points *M*, *N*, and *P*, i.e., the parameters *a*, *b*, and *c*, determine the function’s coefficients impacting the PH characteristics. To describe the model clearly, the 3D schematic view is given in Figure 2, in which the red line represents the function.

The COMSOL Multiphysics 5.5 software is applied for 2D full-wave calculation, and the perfectly matched layer absorbing boundary conditions are applied. The influences of the coordinates of points *M*, *N*, and *P*, and the function coefficients on PH will be discussed below, respectively. The wavelength *λ* of the incident field in the simulation is 632.8 nm, while the diameter and the refractive index of the cylinder are *R* = 7*λ* and *n* = 1.5, respectively.

## 3. Results and Discussion

### 3.1. Changing of y Coordinate of the Upper Intersection M

**Figure 3:**
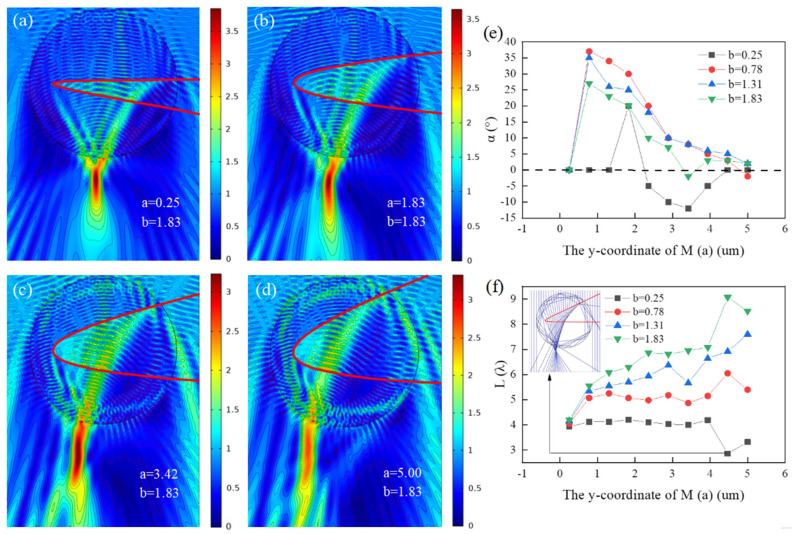
Distribution of electric field under plane wave illumination and the structural property parameters of PH. (**a**–**d**) *P* (−9.00, 0) and *N* (0, −1.83). (**a**) *M* (0, 0.25). (**b**) *M* (0, 1.83) and (**c**) *M* (0, 3.42). (**d**) *M* (0, 5.00). (**e**) The changes of the PH bending angle (*α*) with the upward movement of *M* point from *M* (0, 0.25) to *M* (0, 5.00) under *N* (0, −0.25), *N* (0, −0.78), *N* (0, −1.31), and *N* (0, −1.83) for four cases, respectively. (**f**) Relationship between the effective length (*L*) of PH and the upward movement of *M* point from the same conditions as (**e**). Inset: Ray optical model with *M* (0, 4.47) and *N* (0, −0.25).

In this section, the effect of the upper intersection point *M* on the effective length and bending angle of PH is investigated. When the coordinates of *M* are ranged from (0, 0.25), (0, 1.83), (0, 3.42) to (0, 5.00), the obtained results are shown in Figure 3a–d, respectively, where the red curves represent the function. During the calculations, the lower intersection *N* and the vertex *P* of the function are fixed, the coordinates of which are set as (0, −1.83) and (−9.00, 0). It can be clearly seen that as *M* upshift gradually increases from (0, 0.25) to (0, 5.00), the opening of the depression becomes larger and the energy loss caused by multiple continuous internal reflections inside the depressed cylinder is also enhanced correspondingly, and the optical hook shows a trend of becoming longer and then wider and weaker. Along with the change in the length of the optical hook, the bending angle of the optical hook first becomes larger and then smaller. However, it is worth mentioning that as the upper opening increases, the optical hook shows a more interesting phenomenon of multiple bending with small bending angles, as shown in Figure 3c,d.

In order to better investigate the effect of the opening angle of the function (the opening angle of the depression) on the optical hooks, further studies were conducted for the optical hooks for different lower intersection *N*. Plots of the bending angle and the effective length as a function of the *y* coordinate of *M* are calculated for four different cases of *N* (*b* = 0.25, 0.78, 1.31, 1.83) in Figure 3e,f, respectively. Figure 3e shows the bending angle of the optical hook changing with the *y* coordinate of *M*, while Figure 3f gives the results of the effective length for the same conditions. It can be seen from Figure 3e that the optical hooks produced by the recessed micro-cylinder vary significantly with the change of the intersection points *M* and *N*. The bending angle of the optical hooks generally shows a trend of increasing and then decreasing as the upper intersection point *M* is moved up. When the upshift of point *M* is small, it is easy to obtain a positive bending optical hook bent to the right. When *a* = 3.41 and *b* = 0.78, a bending angle of 36° can be realized. After point *M* moves up to *a* > 2.33, a negative bending optical hook bent to the left may appear, for example, when *b* = 0.25 and *a* = 3.48 in Figure 3e, a negative bending optical hook with *α* = 16° comes out. In addition, a large number of calculations show that it is possible to produce imperfect multiple bending of the depressed cylinder when the upper intersection point *M* is shifted up, such as the secondary bending optical hook in Figure 3c, and the more it is shifted up, the more obvious multiple bending of the optical hook. Regarding the effective length, as shown in Figure 3f, we find that the effective length of the optical hook shows a slow increase with the upward shift of *M*, except for the depression of the lower opening which is relatively small (*b* = 0.25) and has little effect on the propagation direction of the light. In addition, for the same upper intersection *M*, the larger the coordinates of the lower intersection *N* (the lower shift of the *N* point) the larger the effective length of the optical hook and the slight decrease in the effective length can be explained by obvious loss of the outgoing energy resulted from multiple continuous internal reflections inside the micro-cylinder, which is shown in the inserted image of Figure 3f. Therefore, it can be concluded from this section that negative bending PHs and multiple bending PHs can be generated with the increase in the upper opening of the depression.

### 3.2. Changing of y Coordinate of the Lower Intersection N

From the analysis in the above section, it can be seen that the opening angle of the function, i.e., the opening angle of the depression, plays an important role in the regulation of the optical hook, but it is still unclear how to regulate it effectively to obtain a better optical hook and further research is needed. In this section, the optical hooks when the upper intersection *M* is fixed and the position of the lower intersection *N* is changed are studied and analyzed in comparison with Figure 3.

**Figure 4:**
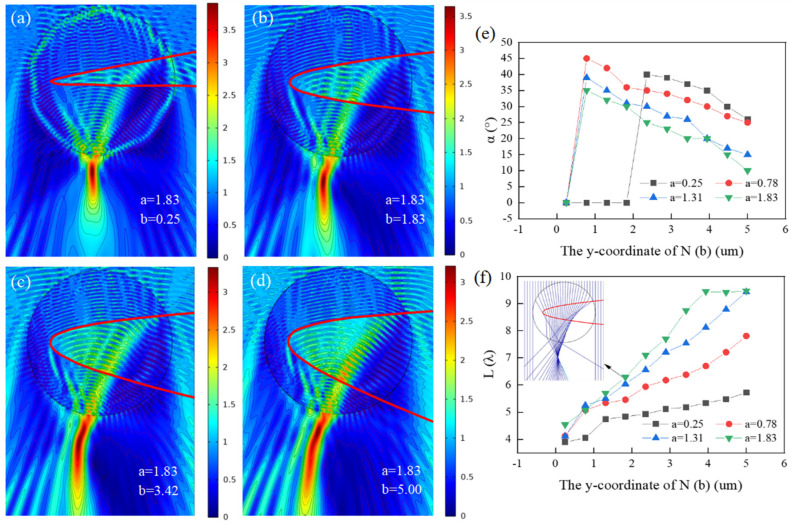
Distribution of electric field under plane wave illumination and the structural property parameters of PH. (**a**–**d**) *P* (−9, 0) and *M* (0, 1.83). (**a**) *N* (0, −0.25). (**b**) *N* (0, −1.83). (**c**) *N* (0, −3.42). (**d**) *N* (0, −5.00). (**e**) Relationship between the bending angle of PH and the downward movement of point *N* from *N* (0, −0.25) to *N* (0, −5.00) in four cases of *M* (0, 0.25), *M* (0, 0.78), *M* (0, 1.31), and *M* (0, 1.83), respectively. (**f**) Relationship between the effective length (*L*) of PH and the downward movement of point *N* from the same range as (**e**). Inset: Ray optical model with *M* (0, 1.83) and *N* (0, −1.83).

Similar to Section 3.1, the effect of the lower intersection point *N* on the effective length and bending angle of PH is researched. When the coordinate of *N* is (0, −0.25), (0, −1.83), (0, −3.42), and (0, −5.00), the result is shown in Figure 4a–d, respectively, with the fixed upper intersection *M* (0, 1.83) and the fixed vertex *P* (−9.00, 0) of the function. From Figure 4a–d, we can see that with the downshift of the lower intersection point *N* from 0.25 to 5.00, i.e., the opening of the depression becomes larger, the optical hook has a longer effective length and the distribution of the intensity becomes more uniform and weaker, and the continuous internal reflections inside the depressed micro-cylinder also decline, leading to a decrease in outgoing energy loss accordingly. In Figure 4a–d, the bending angle of the PH is the largest in Figure 4b, and that can be explained by ray optics in Figure 4f, where the *I_max_* is the inflection point of the PH and the propagation path of light is deflected obviously at the inflection point, resulting in bending of PH. Compared with Figure 3a–d, the depression structure is the same, but the increase in the opening angle of the depression has completely different effects. When the depression increases the upper opening (as in Figure 3a–d), the internal energy loss of the depressed micro-cylinder becomes stronger and the quality of the PH becomes poor, while when the depression enlarges the lower opening (as in Figure 4a–d), the internal energy loss diminishes and the quality of the PH becomes better. The reason for this phenomenon is related to the direction of the beam propagation, i.e., if the beam travels along the y-axis positively, the distribution of the electric field in Figure 4a–d will be the same as that in Figure 3a–d.

In Figure 4e,f, the functions of the bending angle and the effective length with the *y* coordinate of *N* are studied under four different cases of *M* (*a* = 0.25, 0.78, 1.31, 1.83), respectively, where Figure 4e gives the results for the bending angle while Figure 4f shows the results for the effective length in the same condition. As shown in Figure 4e, the bending angle of the optical hook increases first and then decreases almost linearly as the lower intersection point *N* moves down. It can be seen that the bending angle is related to the opening angle of the depression. When *b* > 2.36, with “*a*” changing from 0.25 to 1.83, i.e., the opening angle of the depression becomes larger, the bending angle of the optical hook decreases gradually. By comparing Figure 3e with Figure 4e, it is clear that the optical hook with negative bending can be obtained when the upper intersection point *M* moves up, and when the lower intersection point *N* moves down, the bending angle of the optical hook can be adjusted freely. From Figure 4f, we can see that as the lower intersection point *N* downshift decreases from (0, −0.25) to (0, −5.00) the effective length of the optical hook rises almost linearly apart from the green line (*a* = 1.83). Additionally, during the calculations, we find that when the opening angle is large enough, there is an upper limit for the effective length of the optical hook and when the length reaches its upper limit, no matter how to amplify the opening angle, the length of the optical hook is almost unchanged, except that the start point moves to the left. Further, by comparing Figure 3f with Figure 4f, it can be deduced that raising the lower opening of the depression can prolong the effective length of the PH. So, in this section, we can conclude that in the case of the beam traveling negatively along the *y*-axis, when increases the lower opening of the depression of micro-cylinder, a high-quality PH with a long effective length and uniform light intensity distribution can be obtained. Meanwhile, a smaller opening angle of the depression is more advantageous to obtain a PH with larger bending angle.

### 3.3. Changing of x Coordinate of the Vertex P

**Figure 5:**
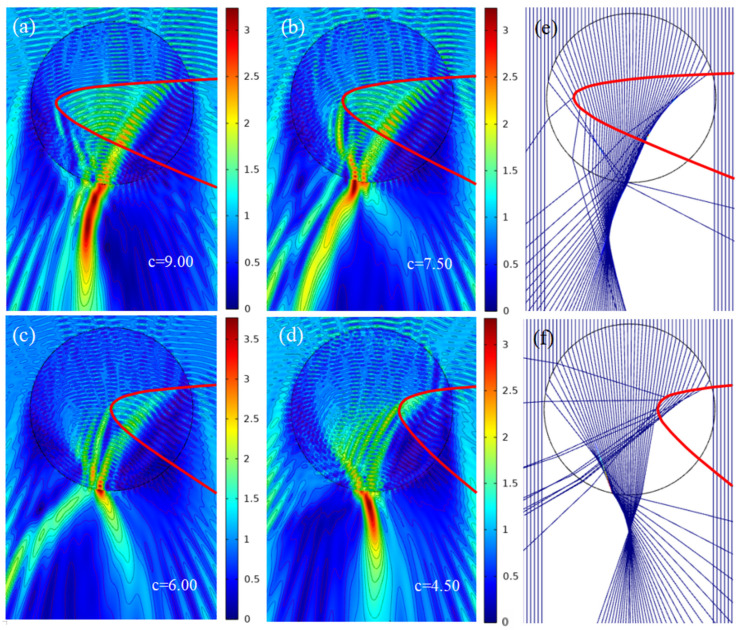
Distribution of electric field and ray optics under plane wave illumination. (**a**) *P* (−9.00, 0). (**b**) *P* (−7.50, 0). (**c**) *P* (−6.00, 0). (**d**) *P* (−4.50, 0). (**e**) Ray optical model with *P* (−9.00, 0). (**f**) Ray optical model with *P* (−4.50, 0). (**a**–**f**) Fixed upper intersection *M* (0, 1.31) and fixed lower intersection *N* (0, −4.47).

Similar to the opening angle of the depression, the depth of the depression will undoubtedly affect the quality of PH. Using the same method, the effect of vertex *P* on the parameters of PH is studied as shown in Figure 5. The field intensity distribution when the coordinate (−c, 0) of *P* is (−9.00, 0), (−7.50, 0), (−6.00, 0) and (−4.50, 0) in turn is shown in Figure 5a–d, respectively. Meanwhile, the results of the ray optics simulation for the two cases of Figure 5a,d are given in Figure 5e,f correspondingly. For the convenience of analysis, we define the shallow opening depth as less than the radius of the micro-cylinder, and the deep opening depth as greater than this radius. We can see that as the vertex *P* moves from inward to outward as shown in Figure 5a–d, the bending direction of the PH transforms from the right (Figure 5a) to the left (Figure 5d) and the effective length of the PH has been modified correspondingly from long (Figure 5a) to short (Figure 5d), while there is almost no PH formed in Figure 5b,c. The formation mechanism of these phenomena can be found in Figure 5e,f. In the case of deep opening depth (as seen in Figure 5e), the direction of light emitting from the micro-cylinder’s shadow side is obviously deflected while almost no deflection occurs in the case of shallow opening depth (as seen in Figure 5f). Meanwhile, a portion of light is consumed during the propagation process in the case of shallow opening depth but not in deep opening depth. As a result, it can be concluded that the position of point *P* (−*c*, 0) handles the bending direction of the PH and the deep opening depth is beneficial to increase the effective length of PH.

### 3.4. Realizing the Adjustable PHs by Controlling Function Coefficients

Based on the above research, we have found that the modulation of the PH can be achieved by changing the coordinates *a*, *b*, and *c* of the depression. Meanwhile, the coordinates *a*, *b*, and *c*, and coefficients *A*, *B*, *C*, and *D* of the SCF have the numerical relationships described in Formulaes (2)–(5) (derived in detail in Appendix A). So, how to adjust coefficients *A*, *B*, *C*, and *D* of the SCF to obtain a demanded or more complex PH? In this part, the tuning of the function coefficients on the PH will be studied in conjunction with the conclusions drawn in Section 3.1, Section 3.2 and Section 3.3 and the Formulaes (2)–(5).

**Figure 6:**
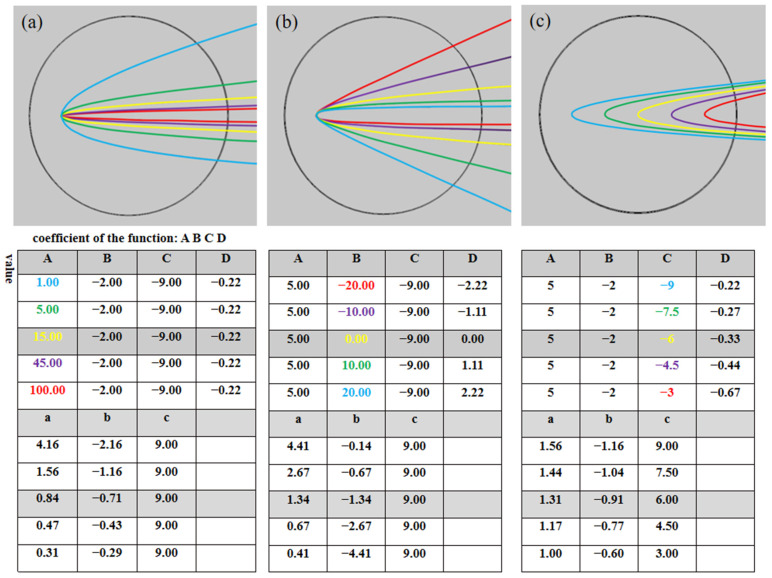
Regulation coefficient *A*, *B*, and *C* control *a*, *b*, and *c* (*M*, *N*, and *P*). (**a**) Change *A*. (**b**) Change *B*. (**c**) Change *C*.

Before discussing the regulation method, the relationships between these coefficients and the opening angle and the depth of the depression are concerned at first, which are shown in Figure 6 in different colors, where Figure 6a–c intuitively give the two-dimensional structure diagrams when coefficients *A*, *B*, and *C* are changed, respectively. Tables inserted below each diagram show the corresponding values of coefficients *A*, *B*, *C*, and *D*, and *a*, *b*, and *c*. As shown in Figure 6a, the smaller coefficient *A* is, the greater the opening angle of the depression is, which denotes that coefficient *A* mainly determines the opening angle of the function. Additionally, it is worth noting that when coefficient *A* is less than 5, the opening angle of the function moves markedly, while when coefficient *A* is larger than 5, the opening angle of the function is modified marginally. Following the conclusion in Section 3.2, in order to achieve a more curved PH, it is necessary to reduce the opening angle, which requires an enhancement in coefficient *A*. Further, it is obvious that the rotation direction of the function is dependent on the coefficient *B* as can be seen from Figure 6b. When coefficient *B* > 0, the curve of the function rotates downward about the vertex as coefficient *B* grows up, and when coefficient *B* < 0, the curve of the function rotates upward about the vertex as coefficient *B* falls down. As stated in Section 3.2, the PH formed by rising the lower opening of the depression has a longer effective length, so it is critical to maintain the coefficient *B* being greater than 0. Similarly, the role of the coefficient *C* is studied in Figure 6c. The greater the absolute value of *C*, the greater the depth of the depression opening. Because “*c*” and *C* have a relation of *C* = −*c*, considering the conclusion in Section 3.3, it can be inferred that the position of the vertex *P* of the function and the opening depth of the depression undoubtedly depend on the coefficient *C*. As for coefficient *D*, it is not found that the influence of coefficient *D* on the PH parameters follows a particular law in our calculation. The feature of coefficient *D* is excluded from this study. So we can say that a high-performing PH can be achieved by changing coefficients *A*, *B*, and *C*, and the specific regulation process of PH by adjusting the coefficients will be detailed based on Formulaes (6)–(8) which are obtained by deformation of Formulaes (2)–(5).

*B* = −*A*(*a* − *b*) (6)

−*C* = *Aab* (7)

*D* = −*B*/*C* (8)

In the regulation process of PH, we have to determine the value of coefficient *C* at first, then substitute the selected coefficient *C* into Equation (7) to obtain the range of coefficient *A*, substitute the selected coefficient *A* into Equation (6) to obtain the value range of coefficient *B*, and then determine the value of coefficient *B*, substitute *B* into Equation (8) the value of coefficient *D* can be finally obtained. The specific regulation process of PH by adjusting the coefficients is as follows.

**Figure 7:**
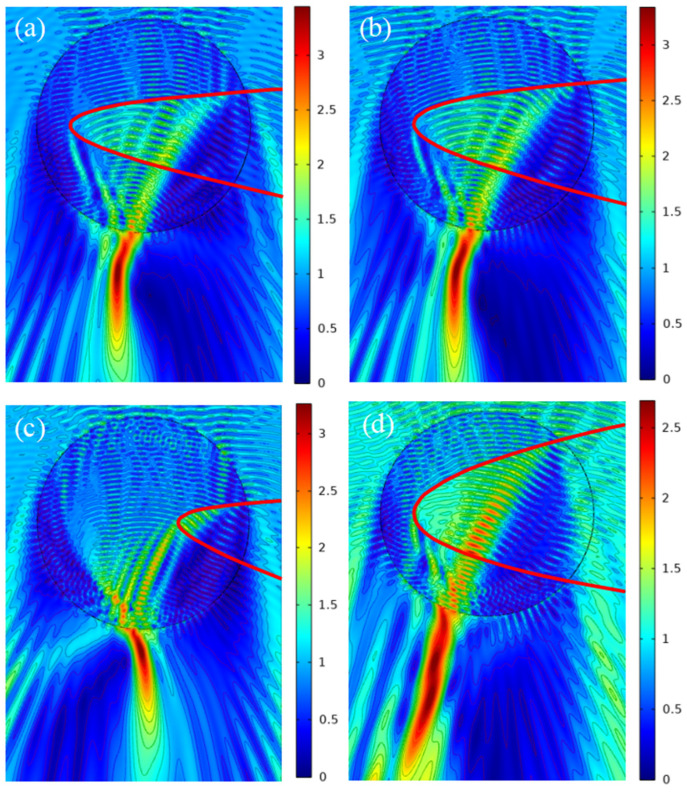
Distribution of electric field for different coefficients *A*, *B*, *C* and *D*. (**a**) *A* = 2.00, *B* = 3.00, *C* = −9.00, and *D* = 0.33. (**b**) *A* = 1.44, *B* = 2.28, *C* = −9.00, and *D* = 0.25. (**c**) *A* = 2.00, *B* = 3.00, *C* = −4.50, and *D* = 0.67. (**d**) *A* = 0.67, *B* = −0.35, *C* = −9.00, and *D* = 0.04.

Firstly, we discuss the coefficient *C*. The range of values for coefficient *C* is restricted by the configuration of the coordinate system and the radius of the micro-cylinder. Based on this point, the coefficient *C* has a value range of (−10.43, −1.57) in our investigation and has been set as −9.00. Due to “*a*” and “*b*” belonging to (0.25, 5.00), the scope of coefficient *A* is (0.36, 144.00) from Formula (7). Substitute the known ranges of “*a*, *b*” and coefficient *A* = 2 into Equation (6) to obtain the interval (−9.50, 9.50) of *B*. Ultimately, based on Formula (8), we obtain the coefficient *D* = 0.33 by taking coefficient *B* = 3.00, and the result is shown in Figure 7a, where the PH formed with an effective length of 7.55*λ* and a bending angle of 17°. (In order to simplify the process of coefficient selection, we present an SCF coefficient selection procedure with respect to the data in this paper in Appendix B.) If additional parameters for the PH are required, multiple types of PH may be developed by performing the procedure outlined above. As shown in Figure 7b we obtain the high-quality PH bending to the right, whose effective length *L* = 8.72*λ* and bending angle *α* = 15°. The PH bending to the left with *L* = 5.96*λ* and *α* = −14° is realized in Figure 7c. Furthermore interesting is when *A* = 1.44, *B* = 2.28, *C* = −9.00, and *D* = 0.25, the PH with three bending times can be obtained as shown in Figure 7d, and its total effective length reaches almost 11.90*λ*. The bending angle and the effective length of PH obtained by the proposed method are compared with other data in the existing literature, as shown in Table 1. It can be obviously seen that the controllable range of the bending angle and the effective length of the PH increases through our method. So, in this part, we achieve the valid control of the characteristic parameters of the PH through the coefficient regulation of the function, and the various parameters of PH such as longer or shorter effective length, different bending directions and multiple bending properties have been also carried out. Simple function regulation provides a new fashion for the manipulation of the PH. In addition, it is worth noting that the simulation results in this paper use the physical field to control the grid. By adjusting the grid accuracy, we find that the simulation results obtained are consistent whether the grid size is coarser or refined. Therefore, the law discussed above is not sensitive to grid accuracy.

The concave micro-columns proposed in this work can be fabricated using the femtosecond laser direct writing technology. At present, ultra-fast laser direct writing technology can realize the precise and controllable circular section matching of polymethyl methacrylate PMMA material, and the etching aperture is up to 250 nm [43,44]. At the same time, the traditional femtosecond laser direct writing device has a high-precision XYZ electric platform, and the platform stepping accuracy can reach 0.04 μm [45] (*x*, *y* axis). A number of controllable holes are connected to form a hollow channel, usually using a hole formed by a femtosecond laser in a transparent material. The method can also be used to fabricate micro-fluidic devices, micro-electrodes, and micro-integrated chips.

## 4. Conclusions

This is the first time that a novel method to generate PHs is proposed by introducing the SCF to an irregular structure of a concave micro-cylinder. The characteristic parameters of PH, such as the effective length, bending angle, and bending direction, can be effectively modulated by changing the function coefficients *A*, *B*, and *C*, which determines the opening angle, the rotation direction, and the opening depth of the function, respectively. PHs of micro-nano structures with cylindrical-like concaves were modulated effectively with varying function coefficients and calculated by the finite element method. During the research, we found that it is easy to generate a long effective length of PH by enlarging the opening depth and the lower opening of the SCF, to produce a curvy PH through SCF with a small opening angle, and to achieve a multiple bending or negative bending PH by increasing the upper opening of the SCF. All of the principles can also be suitable for other cylindrical-like micro-nano structures. It is worth noting that the effective length and the bending angle of the high-performance photonic hooks can reach 11.90*λ* and 46°, respectively. Further, we must point out that the function used in our research only with respect to the requirement of the surface description of cylindrical-like concave structure, so other different types of functions can also be produced in specific studies. The obtained results and the method provided here greatly expand the generation and regulation approaches of PH and provide potential applications in beam controlling, micro-particle capture, manipulation of living cells [46], and electromagnetic-wave-direction manipulation [47,48,49,50].

## Figures and Tables

**Table 1:** Comparison of the bending angle and the effective length of the PH between the proposed method and previous work.

Structure Type of the Particle	Method	The Range of the Bending Angle (°)	The Range of the Effective Length
Regular Structure	Asymmetry illumination	No data	No data
	Asymmetry Materials	(−7.00, 29.70)	About 4.20~7.20 μm
	Asymmetry Structural	(6.00, 22.80)	About 1~3.00 μm
Irregular Structure	Structure-constrained function	(−12.00, 46.00) and multiple bending	7.53 μm

## Data Availability

Data is available upon request from the corresponding author.

## Appendix A

The structure constraint function in our study begin with a general parabola given as:

(A1) $$x=Ay^{2}+By+C$$

Under the premise that the curve is symmetric about the *x*-axis and its vertex is on the *x*-negative semi-axis (on-axis case), the upper intersection, lower intersection and the vertex of the curve with the coordinate axis are (0, *a*), (0, −*b*), and (−*c*, 0), respectively. (where *a* > 0, *b* > 0, *c* > 0 and *a* = *b*). It is possible to alter the position of the three intersections by changing *A*, *B*, and *C*, and their relationship is shown in (A2).

(A2) $$\begin{array}{l} A=\frac{c}{a^{2}}\\ B=\frac{2c}{a}\\ C=-c \end{array}\begin{array}{l} a=\frac{B}{2A}\\ b=a=\frac{B}{2A}\\ c=-C \end{array}$$

Considering the asymmetric case of the curve, we add a “*Dxy*” term to the original Equation (A1).

(A3) $$x=Ay^{2}+By+C+Dxy$$

Separating variables “*x*” and “*y*”, the Equation (A3) can be transformed into (A4)

(A4) $$x=\frac{Ay^{2}+By+C}{1-Dy}$$

When *D* is small, the curve maintains its original shape but no longer has *x*-axis symmetry. At this time the relationship between *A*, *B*, and *C* and *a*, *b*, and *c* changes to (A5). (*A* > 0, *C* < 0 and *B*/*D* > 0)

(A5) $$\begin{array}{l} A=\frac{c}{ab}\\ B=-\frac{(a-b)c}{ab}\\ C=-c\\ D=-\frac{\left(a-b\right)}{ab} \end{array}\begin{array}{l} \;\\ a=\frac{\sqrt{B^{2}-4AC}+B}{2A}\\ b=\frac{\sqrt{B^{2}-4AC}-B}{2A}\\ c=-C \end{array}$$

So far, the structural constraint function has been successfully established and the opening angle, opening depth, and rotation direction of the function can be effectively controlled by adjusting coefficients *A*, *B*, *C*, and *D*. Due to the ordinate of the function vertex being on the *x*-negative semi-axis, we regard this situation as on axis.

Now discussing the off-axis case, keep the upper and lower intersections (0, *a*) and (0, −*b*) of the curve unchanged, and the vertex coordinates become (−*c*, *d*), denoting that the vertex can move up and down relative to the *x*-axis. Still starting with the symmetric parabola (A1), the connection of *A*, *B*, and *C* and *a*, *b*, *c*, and *d* become (A6).

(A6) $$\begin{array}{l} \;\\ A=\frac{4c}{{\left(a+b\right)}^{2}}\\ B=-\frac{4(a-b)c}{{\left(a+b\right)}^{2}}\\ C=-\frac{4abc}{{\left(a+b\right)}^{2}} \end{array}\begin{array}{l} a=\frac{\sqrt{B^{2}-4AC}-B}{2A}\\ b=\frac{\sqrt{B^{2}-4AC}+B}{2A}\\ c=\frac{B^{2}-4AC}{4A}\\ d=\frac{a-b}{2}=-\frac{B}{2A} \end{array}$$

It can be seen that the form of the equation is much more complicated than the original, and then considering the asymmetric case, the form of the initial equation remains (A3).

The relationship between the coefficients of the function becomes complex, and in order to facilitate the representation of *A*, *B*, *C*, and *D*, the form of *D* can be given first by simplification.

(A7) $$D=-\frac{a-b-2d}{ab+d^{2}}$$

The relationship of the remaining coefficients at this point is (A8).

(A8) $$\begin{array}{l} A=-\frac{c(1-Dd)}{\left(d-a)(d+b\right)}\\ B=\frac{c(1-Dd)(a-b)}{\left(d-a)(d+b\right)}\\ C=\frac{abc(1-Dd)}{\left(d-a)(d+b\right)} \end{array}$$

With respect to *a*, *b*, *c*, and *d*, the relationship between *a*, *b*, and the coefficients can be derived directly.

(A9) $$\begin{array}{l} a=\frac{\sqrt{B^{2}-4AC}-B}{2A}\\ b=\frac{\sqrt{B^{2}-4AC}+B}{2A} \end{array}$$

Combining (A7) with the (A9) and substituting d into the (A4), the relationships between *c* and *d* and *A*, *B*, *C*, and *D* can be conveniently expressed as:

(A10) $$\begin{array}{c} c=-\frac{Ad^{2}+Bd+C}{1-Dd}\\ d=\frac{1}{D}(1-\sqrt{1+\frac{BD+CD^{2}}{A}}) \end{array}$$

At this moment, the off-axis equation and the relationship between coefficient *A*, *B*, *C*, and *D* and *a*, *b*, *c*, and *d* is successfully established.

It is worth noting that although the off-axis case is discussed, we only utilize the equation for the on-axis situation in the actual simulation since the coefficient *D* has no regular effect on the PH’s properties.

## Appendix B

This procedure is realized by MATLAB software, and the program content is as follows:

*C_min_* = −10.43;

*C_max_* = −1.57;

prompt = sprintf(“please enter *C*(%4.2*f*,%4.2*f*):”,*C_min_*,*C_max_*);

*C* = input(prompt)

*A_min_* = C/5/5;

*A_max_* = −C/0.25/0.25;

prompt = sprintf(“please enter *A*(%4.2*f*,%4.2*f*):”,*A_min_*,*A_max_*);

*A* = input(prompt)

*B_min_* = −4.75 × *A*;

*B_max_* = 4.75 × *A*;

prompt = sprintf(“please enter *B*(%4.2*f*,%4.2*f*):”,*B_min_*,*B_max_*);

*B* = input(prompt)

*D* = −*B*/*C*;

*A*

*B*

*C*

*D*

*f*1 = @(*x*,*y*) ( *x*+ 6)^2^ + *y*^2^ − (0.6328 × 7)^2^;

fimplicit(*f*1)

hold on

*f*2 = @(*x*,*y*) *A* × *y*_2_ + *B* × *y* + *C* + *D* × *x* × *y* − *x*;

fimplicit(*f*2);

axis([−12,0,−6,6])
